# Combined plasma rich in growth factors and adipose-derived mesenchymal stem cells promotes the cutaneous wound healing in rabbits

**DOI:** 10.1186/s12917-018-1577-y

**Published:** 2018-09-21

**Authors:** Deborah Chicharro, Jose M. Carrillo, Mónica Rubio, Ramón Cugat, Belén Cuervo, Silvia Guil, Jerónimo Forteza, Victoria Moreno, Jose M. Vilar, Joaquín Sopena

**Affiliations:** 1 Bioregenerative Medicine and Applied Surgery Research Group, Animal Medicine and Surgery Department, Veterinary Faculty, Universidad Cardenal Herrera-CEU, CEU Universities, Valencia, Spain; 2 Orthopaedic service in Hospital Quirón, Artroscopia GC, Barcelona, Spain; 3 Anatomy and Comparative Pathology Department, Universidad de Córdoba, Córdoba, Spain; 4 Molecular Pathology and Translational Research in Oncology, Centro de Investigación Príncipe Felipe, Valencia, Spain; 5 Neuronal and Tissue Regeneration Laboratory, Centro de Investigación Príncipe Felipe, Valencia, Spain; 6 Animal Pathology Department, IUIBS, Universidad de Las Palmas de Gran Canaria, 35416, Transmontaña s/n, Arucas, Las Palmas, Spain

**Keywords:** Adipose-derived mesenchymal stem cells (ASCs), Plasma rich in growth factors (PRGF), Wound healing, Rabbits, Skin, Regenerative medicine, Growth factors

## Abstract

**Background:**

The use of Plasma Rich in Growth Factors (PRGF) and Adipose Derived Mesenchymal Stem Cells (ASCs) are today extensively studied in the field of regenerative medicine. In recent years, human and veterinary medicine prefer to avoid using traumatic techniques and choose low or non-invasive procedures. The objective of this study was to evaluate the efficacy of PRGF, ASCs and the combination of both in wound healing of full-thickness skin defects in rabbits. With this purpose, a total of 144 rabbits were used for this study. The animals were divided in three study groups of 48 rabbits each depending on the administered treatment: PRGF, ASCs, and PGRF+ASCs. Two wounds of 8 mm of diameter and separated from each other by 20 mm were created on the back of each rabbit: the first was treated with saline solution, and the second with the treatment assigned for each group. Macroscopic and microscopic evolution of wounds was assessed at 1, 2, 3, 5, 7 and 10 days post-surgery. With this aim, 8 animals from each treatment group and at each study time were euthanized to collect wounds for histopathological study.

**Results:**

Wounds treated with PRGF, ASCs and PRGF+ASCs showed significant higher wound healing and epithelialization rates, more natural aesthetic appearance, significant lower inflammatory response, significant higher collagen deposition and angiogenesis compared with control wounds. The combined treatment PRGF+ASCs showed a significant faster cutaneous wound healing process.

**Conclusions:**

The combined treatment PRGF+ASCs showed the best results, suggesting this is the best choice to enhance wound healing and improve aesthetic results in acute wounds.

## Background

Wound healing is a complex and dynamic process that includes inflammation, tissue formation and remodeling, involving the interaction of multiple cell types, cytokines, growth factors, and chemokines [1, 2]. When the physiological mechanisms of wound healing are interrupted, chronic or non-healing wounds can appear [3]. A structured assessment of the wound healing should include basic components of the healing process such as inflammatory response evaluation, angiogenesis, fibroplasia and epithelialization [4]. The importance of evaluating macroscopic scar quality has also been highlighted by other authors [5].

Regenerative skin wound therapy is a novel and rapidly developing field of biomedical research that aims to promote wound healing [6] and focuses on replacing, restoring and regenerating damaged cells, tissues and organs [7].

Stem cells, with their properties to self-renew and undergo differentiation are being extensively assessed for their wound healing potential [8]. Adipose-derived stem cells (ASCs) are an appealing source of mesenchymal stem cells (MSCs) due to their abundant availability, good expansion capacity, their ability to proliferate in culture, and cryopreservation capacity [9]. They have been already successfully used in the treatment of soft tissue defects, scars, and burn injuries by showing an acceleration and improvement on the quality of the wound healing process [10].

ASCs promote angiogenesis, epithelial migration, secretion of growth factors and differentiate into multiple lineages [11]; thus, enhance the wound healing process with less scar formation. The exact mechanism of action is still under investigation. It is postulated that ASCs can stimulate tissue regeneration by differentiating into epithelial cells or by secreting paracrine factors to activate endogenous repair mechanisms [12].

Blood platelets are a natural source of growth factors and cytokines that help accelerating the normal wound healing process [13]. The main growth factors which are responsible for promoting the re-epithelialization process are EGF, FGF-2, IGF-1 and TGFα [14]. These growth factors trigger biological effects such as cell migration, angiogenesis, cell proliferation and differentiation, promote extracellular production and inflammation resolution. These are key elements in the tissue repair process [15]. Plasma Rich in Growth Factors (PRGF) is a portion of autologous plasma enriched of proteins and circulating growth factors with a platelet concentration above baseline [16]. The advantages and merits of PRGF are apparent since it is a simple, cost-effective and safe product [9]. Successful clinical applications have been reported using these Platelet Rich Plasma (PRP) derivates in wound and soft tissue repair [17], cosmetic surgery [18], nervous tissue repair [19], orthopaedics [20] and chronic ulcers [21].

A synergetic effect is suggested when ASCs and PRGF are used together, where growth factors act as vehicles and potentiators of MSCs [22, 23].

Based on this, the aim of the study is to compare the efficacy of using PRGF, ASCs and the combination of both and assess a possible synergistic effect in acute full-thickness cutaneous wounds in a rabbit model.

## Methods

### Animals

A total of 144 adult female New Zeland rabbits (8 months old), 48 animals per treatment group (PRGF, ASCs or PRGF+ASCs) and therefore 8 animals per study time (1, 2, 3, 5, 7 and 10 days) with an average weight of 3152 g were used to carry out a prospective randomized experimental study. Animals were housed in spacious individual cages with food and water ad-libitum and were monitored daily for signs of discomfort, infection and weight loss. An acclimatization period of 7 days was established before starting the experiment to allow animal adaptation. Complete physical examination, haematology and serum biochemical analyses were also performed and results were within normal reference range values.

This study was approved by the Ethics Committee of Animal Welfare (CEBA) of the university CEU Cardenal Herrera of Valencia (Spain) in accordance with European legislation (86/609/CEE) .

### Plasma rich in growth factors (PRGF) preparation

**Fig. 1:**
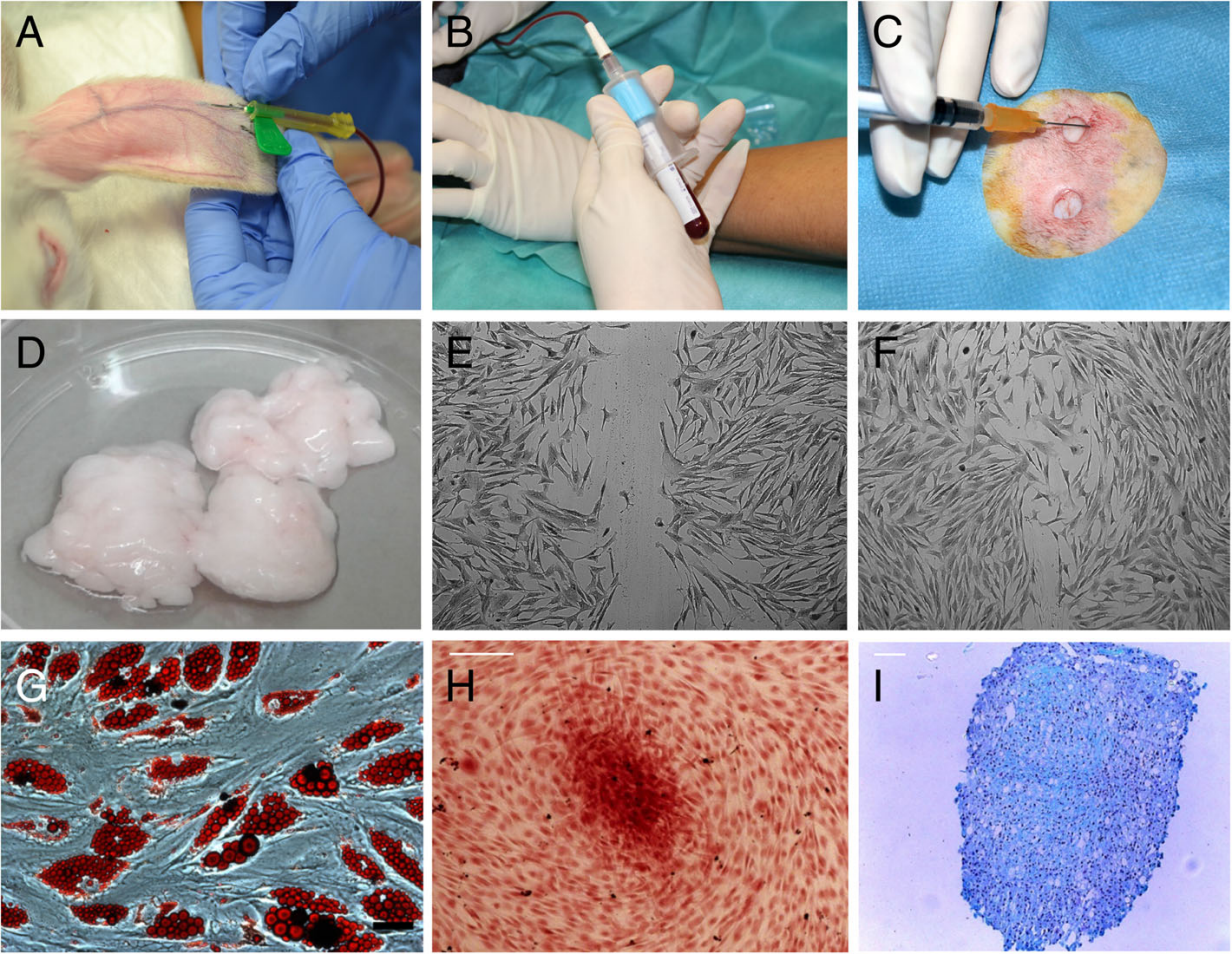
Preparation and infiltration of Plasma Rich in Growth Factors (PRGF) together with Adipose-Deived Mesenchymal Stem Cells (ASCs) isolation, culture and differentiation. **a** Blood extraction from auricular artery of each rabbit (5 ml). **b** Blood collection in vacutainer sodium citrate 3,8% tubes (BTI Biotechnology institute, Álava, Spain). **c** Perilesional PRGF infiltration into corresponding cutaneous wounds. **d** Harvested adipose tissue from donor rabbit. **e**, **f** Morphology of ASCs cultured in vitro, migration and wound closure capacity in vitro. **g**, **h**, **i** Multipotent ability of ASCs by induced differentiation of adipocytes (G) is shown by Oil Red staining, scale bar: 15 μm osteoblasts (H) showing calcium accumulation by Alizarin Red staining, scale bar: 100 μm; and chondrocytes (I) showing high content of condroitinsulfate enriched matrix stained by Alcian Blue, scale bar: 100 μm

PRGF®-Endoret® technology was used to obtain an autologous preparation of PRP. A total of 5 ml of blood was collected from the auricular artery of each rabbit under sedation with intramuscular dexmedetomidine (10 μg/kg), ketamine (20 mg/kg) and morphine (0.2 mg/kg) and under sterile conditions in vacutainer sodium citrate 3.8% tubes (Blood collecting tubes®, BTI Biotechnology institute, Álava, Spain). The tubes were centrifuged at 460 g for eight minutes (PRGF® System III, Biotechnology Institute®, Álava, Spain) to separate the different blood phases. Two sterile fractionation tubes were used to collect PRGF and the Plasma Poor in Growth Factors (PPGF), with sterile pipettes of 200 μl and 1000 μl respectively. The 0.5 ml fraction located immediately above the buffy coat corresponded to PRGF. Just before cutaneous infiltration to activate platelets for growth factors release, 10% calcium chloride was added to PRGF (50 μl/ml of PRGF) (Fig. 1a, b, c).

### Adipose-derived mesenchymal stem cell (ASCs) isolation, culture and differentiation

Allogenic ASCs were used from an individual donor rabbit coming from the same institution. Under general anesthesia and sterile conditions, a total of 31.3 g of inguinal adipose tissue was collected. Additionally, a 20 ml blood sample was obtained from the external jugular vein into blood collection tubes without anticoagulant (BD Vacutainer®, Plymouth, UK). Both samples were transported to the Research Center Príncipe Felipe (CIPF) in an enclosed package at 4 °C where ASCs isolation and cell culture was carried out.

At laboratory, adipose tissue was washed with Phosphate Buffered Saline Solution (PBS) plus antibiotics. Total collected fat was distributed into ten grams of adipose tissue for enzymatic reaction, by incubating in a PBS solution containing penincilin, streptomycin (Gibco 15,140) and collagenase (0.07% Sigma C9891 CA, USA). The tissue was then manually cut into small pieces and digested overnight at 37 °C. The following day, the digested adipose tissue was washed and the resulted stromal vascular fraction was cultured in autologous serum allowing cells to grow until one million cells per gram were obtained and subjected up to three consecutive passages before cell transplantation. For tissue transplantation, one million cells were suspended in growth medium using 10% rabbit serum.

For induced differentiation, ASCs after passage 4 were subjected, to induce adipogenesis, osteogenesis and chondrogenesis. Briefly, adipogenesis was induced in confluent ASC culture for 12 days in the presence of differentiation medium (Adipose Derived stem cell Basal Medium; Lonza Co). The adipogenic differentiation was evaluated by Oil Red staining of the lipid vacuoles in formalin fixed cultures; For *osteogenesis*, ASC were seeded in coated plates in medium containing 0.1 μM dexamethasone, 50 μM Asc2P and 10 mM μ-glycerophosphate (Osteogenic Basal Medium; Lonza Co.) with 10% FBS for 4 weeks. For detection of extracellular calcium deposits Alizarin Red staining was used in formalin fixed cultures; Alizarin Red Solution (0.2 g/L water) was incubated for 2–3 min, until the reaction was observed microscopically. *Chondrogenesis was induced* from ASC in “Micromass” in the presence of TGF-β 1 and 3 (10 ng/ml), Asc 2P (50 μM) and insulin (6.25 μg/ml) (Chondro BulletKit; Lonza Co.) for 4 weeks. Alcian blue (0.1 g/L in water, pH 1.0) was used to detect the presence of enrichment of sulphated proteoglycans in the extracellular matrix. (Fig. 1d, e, f, g, h, i).

### Wound model and treatments

Rabbits were premedicated with dexmedetomidine (10 μg/kg; Dexdomitor®, Esteve, Spain), ketamine (20 mg/kg; Imalgene, Merial, Spain) and morphine (0.2 mg/kg; B-Braun, Germany). The dorsal thoracolumbar area of rabbits was clipped and prepared for aseptic surgery. General anesthesia was mask induced and mantained with sevoflurane (Sevoflo®, Esteve, Spain).

Two full-thickness wounds of 8 mm in diameter and separated 20 mm from each other were created on the dorsal thoracolumbar area of each rabbit using disposable dermal biopsy punches (Kruuse, UK); one for the placebo treatment (saline solution), and the other for the study treatment (PRGF, ASCs, or PGRF+ASCs). Each wound was treated injecting perilesionally with 0.1 ml of the corresponding treatments; ASCs preparations contained at least 1 × 10^6^cells. Cefovecin as prophylactic antibiotic and buprenorphine during 3 days was administered. Rabbits were then randomly divided into six groups according to their survival time (1, 2, 3, 5, 7, and 10 days after wounding), subsequently a total of 8 animals from each treatment group and at each study time (1, 2, 3, 5, 7, and 10 days after wounding) were euthanised and wounds were evaluated macroscopically and histologically. Study times were chose based on a preliminary study in rabbits not published yet were a significant acceleration of the wound healing process was found at day 7 but not at day 14. In the same way, other authors also observed significant differences during the early healing period [13, 24].

### Macroscopic study

Rabbits were euthanized following spanish royal decree 53/2013 with an overdose of intracardiac pentobarbital. Immediately after sacrifice, a macroscopic assessment of each wound at the different study times was made by two blinded investigators. Thus, good quality photographs were taken for subsequent digital study.

Wound closure was calculated by measuring the largest (original size) and smallest (open wound area) diameters of the wounds as a wound healing percentage ((largest diameter-smallest diameter/largest diameter)*100). Additionally, based on Oppenheimer et al. [5] semi-quantitative scale, scar quality was evaluated based on color (1:hiperpigmentated, 2:pigmented, 3:red, 4:almost-normal, 5:normal), thickness (1:keloid, 2:hyperthrophic, 3:almost-normal, 4:normal), and wound retraction (1:very retracted, 2:mild retraction, 3:no retraction). Infection was categorized as a yes or no condition based on the presence or abscense of abcess or discharge of some kind of exudates such as seropurulent, haemopurulent or pus.

### Tissue processing, sectioning and staining

For histopathological study, tissues from every study time were fixed in 4% formalin for 24 h, prior to processing for paraffin embedding. Sections of 5 μm containing the entire wound area were obtained and stained with H&E for analysis of re-epithelialization and inflammatory infiltrate intensity and Masson’s trichome staining was used to assess the degree of collagen deposition and angiogenesis.

The stained sections were digitalized with the use of a photomicroscope and an attached digital camera, and the histological images were transferred to a computer equipped with image analysis software (Image Pro-Plus®, Media Cybernetics, USA) to perform quantitative measurements by two blinded pathologists.

### Microscopic study

In H&E stained sections, a representative histological image including the entire wound and surrounding healthy tissue was captured using Pannoramic Viewer software (3DHISTECH) to calculate the epithelialization rate percentage. The epithelial gap (distance between the advancing edges of epithelium) and the original histological wound distance (thicker epithelium) was measured with the freehand tool to calculate the percentage of re-epithelialization ((original distance-epithelial gap/original distance)*100). Moreover, inflammatory infiltrate intensity and distribution was also evaluated with H&E stained sections. Entire dermis was evaluated based on a semi-quantitative scale published by Lowry et al. [25] for intensity inflammatory infiltrate assessment (0:no infiltration, 1:mild infiltration, 2:strong infiltration, 3:severe infiltration) and distribution (1:focused beneath epidermis, 2:diffuse beneath epidermis, 3:Both).

In Masson trichrome stained sections, six histological images per slide were captured (three superficial and three deep dermis). Angiogenesis was quantified by direct counting of blood vessels based in the average of the six histological captures. To asses the percentage of collagen deposition, mature collagen (dark blue) was determined via quantitative morphometry using Image Pro Plus software, by transforming the dark blue color to black and white pictures, where the white area corresponded to the mature collagen, which was expressed as the percentage of pixels of positive colllagen staining divided by total pixels of the image.

### Statistical analysis

A descriptive study of the mean, standard deviation and confidence intervals was made for each variable. A value of *p* < 0.005 was considered significant. Nonparametric Kruskal-Wallis tests were used to compare non-normally distributed variables and an ANOVA test for variables which followed a normal distribution. Normality of data was tested in every quantitative variable with Shapiro-Wilk test and variance homogeneity with Levene test.

The data were processed using the SPSS 20.0 programm for Mac (SPSS®Inc., Chicago, USA).

## Results

### Wound closure and epithelialization rate evaluation

**Fig. 2:**
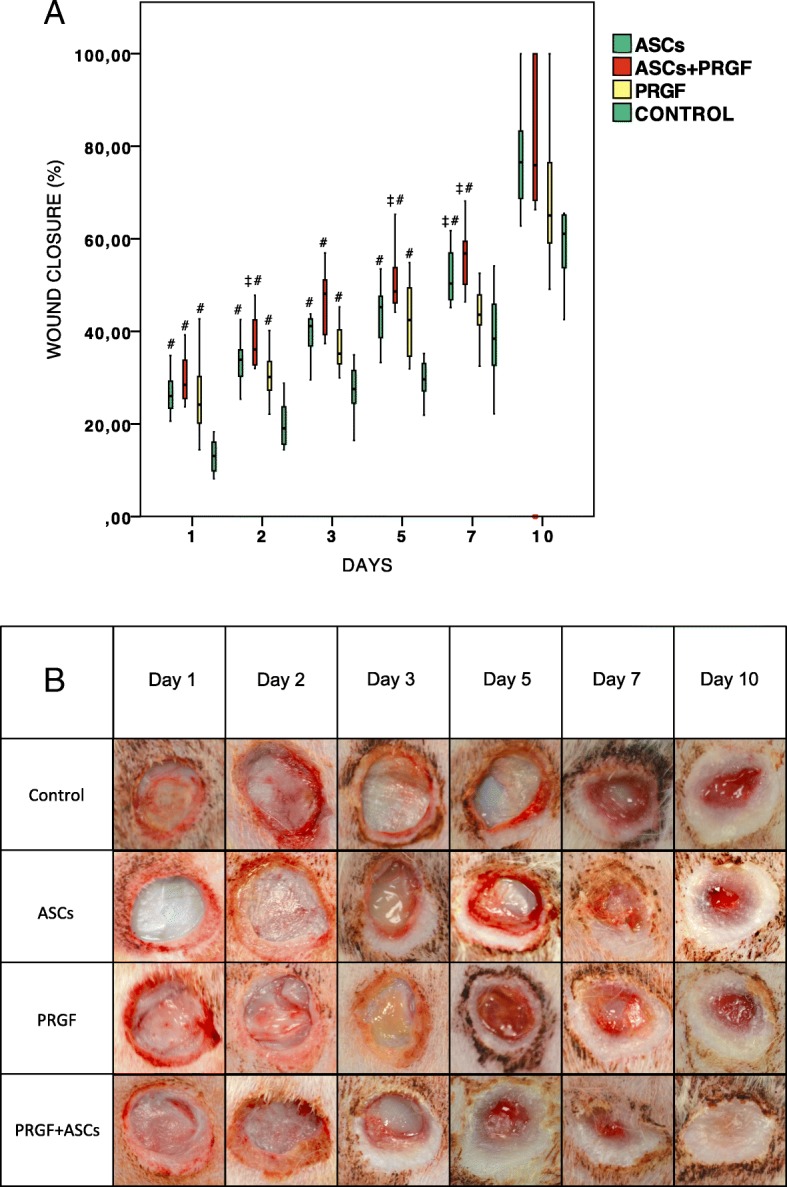
Macroscopic wound healing evaluation. **a** Wound closure rates: Comparison in between treatment groups regarding wound closure percentage in each of the studied times. Statistical significant differences between groups are shown with the following: + (ASCs group), † (ASCs+PRGF group), ‡ (PRGF group), # (CONTROL group). **b** Representative photographs of cutaneous wounds from each treatment group at the different studied times

Wounds treated with PRGF, ASCs or combination of both showed significant faster wound healing rates compared to control wounds throughout the study except at day 10 (*p* < 0.001). When single therapies were compared, ASCs demostrated at day 7 to accelerate the wound healing process more efficiently than PRGF (*p* < 0,001) (Fig. 2). Additionally, the ASCs+PRGF group demonstrated significant faster wound closure at days 2, 5 and 7 compared with PRGF (*p* < 0.001).

**Fig. 3:**
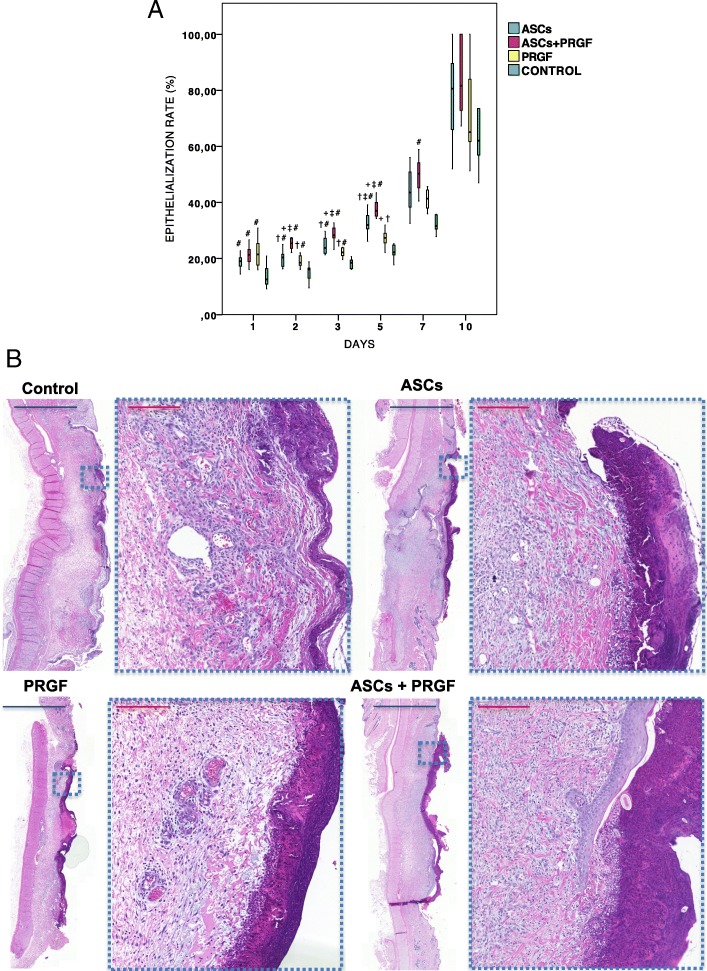
Microscopic wound healing epithelialization rate evaluation. **a** Epithelialization rate: Comparison in between treatment groups regarding epithelialization rate percentage in each of the studied times. Statistical significant differences between groups are shown with the following: + (ASCs group), † (ASCs+PRGF group), ‡ (PRGF group), # (CONTROL group). **b** Hematoxylin eosin staining. Results from a representative animal within the different treatment groups. Histological images show representative evaluated samples (Blue scale = 2 mm; Red scale = 0,2 mm)

Histological analysis of wounds showed as, in the same manner, ASCs+PRGF treated wounds presented faster epithelialization rates at days 2, 3 and 5 (*p* < 0.001) compared to PRGF, ASCs and control wounds (Fig. 3).

### Aesthetic assessment of wounds

No statistical significant differences were observed regarding scar retraction and infection variables. However, scar color and thickness tended to be almost or completely normal in PRGF, ASCs and ASCs+PRGF treated wounds compared with control except for significant better results at days 2 and 3 in scar color and thickness in ASCs and ASCs+PRGF treated wounds (*p* < 0.05).

### Histological inflammatory cell infiltration and distribution evaluation

The observed acute inflammatory infiltrate was mainly composed of neutrophils with an irregular fibrous connective tissue during the first two days. In addition, some macrophages and congestive blood vessels were also evident within the dermis 2–3 days after injury.

**Fig. 4:**
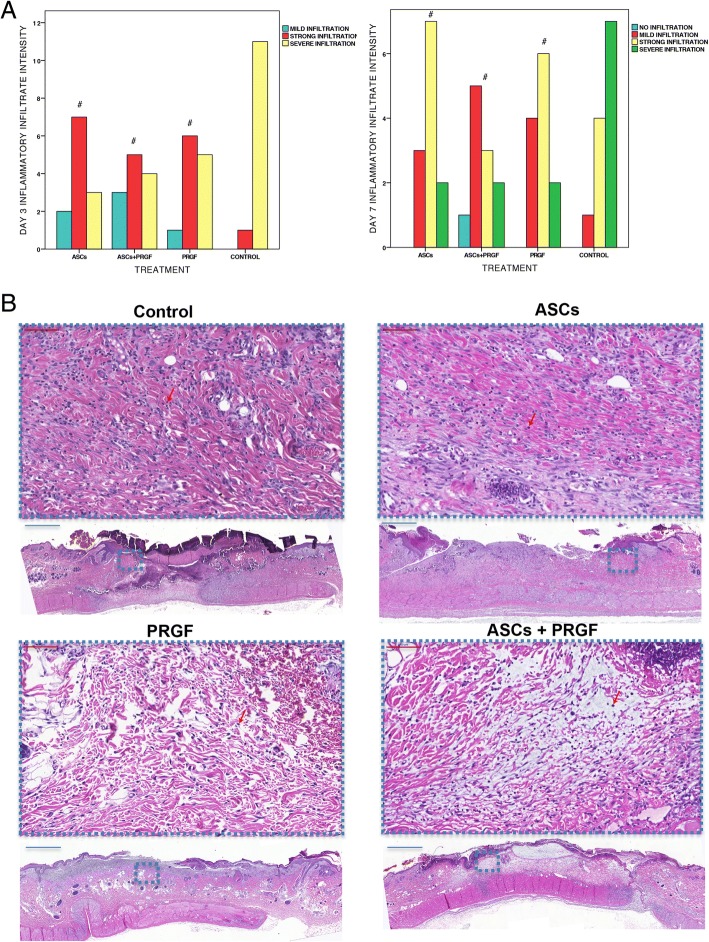
Microscopic wound healing inflammatory cell infiltration evaluation. **a** Evaluation of inflammatory cell infiltration intensity: Comparison in between treatment groups regarding inflammatory cell infiltration intensity at representative studies times (Day 3 and 7). Statistical significant differences between groups are shown with the following: + (ASCs group), † (ASCs+PRGF group), ‡ (PRGF group), # (CONTROL group). **b** Hematoxylin eosin staining. Results from a representative animal within the different treatment groups. Histological images show representative evaluated samples (Blue scale = 1 mm; Red scale = 0,2 mm)

A significant decreased inflammatory cell infiltration in all study groups compared with control was observed at days 1, 2, 3 and 7 (*p* < 0.05); in addition, a larger number of animals showed a mild or absent inflammatory infiltrate in ASCs+PRGF group compared with the other treatments throughout the study (Fig. 4). No statistical significant differences were shown regarding inflammatory cell infiltrate distribution throughout the study (*p* > 0.05).

### Wound healing angiogenesis assessment

**Fig. 5:**
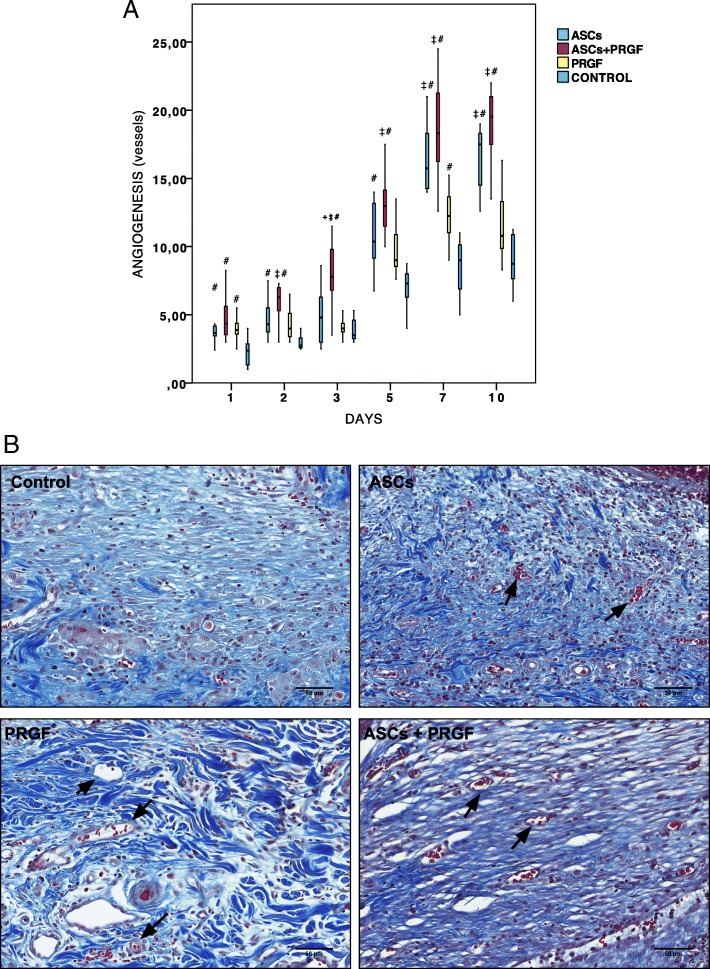
Microscopic wound healing angiogenesis assessment. **a** Quantification of new blood vessels: Comparison in between treatment groups regarding angiogenesis in each of the studied times. Statistical significant differences between groups are shown with the following: + (ASCs group), † (ASCs+PRGF group), ‡ (PRGF group), # (CONTROL group). **b** Masson trichrome staining. Results from a representative animal within the different treatment groups. Arrows show representative new blood vessels formed (Scale bar = 10 mm)

Masson trichrome staining revealed a significant increase in blood vessel formation in ASCs, PRGF and ASCs+PRGF treated wounds at all studied days compared to control groups (*p* = 0.010, *p* = 0.001, *p* = 0.001). ASCs+PRGF treated wounds produced 2–3 fold more blood vessels than placebo, PRGF and ASCs groups, with a maximal increase on day 7 (Fig. 5). These results suggest that ASCs+PRGF stimulates angiogenesis in an early phase of the wound healing process; Thus, PGRF+ASCs treatment showed a synergistic response in terms of angiogenesis.

The ASCs treated wounds showed a significant greater blood vessel density compared to control groups at all studied times except for day 3. Additionally, with respect to PRGF group they also demonstrated higher wound vascularity on days 7 and 10. Moreover, a significant increase in the number of blood vessels was noticeable in the PRGF treated wounds compared to control groups at days 1 and 7 after wounding.

### Collagen deposition assessment

**Fig. 6:**
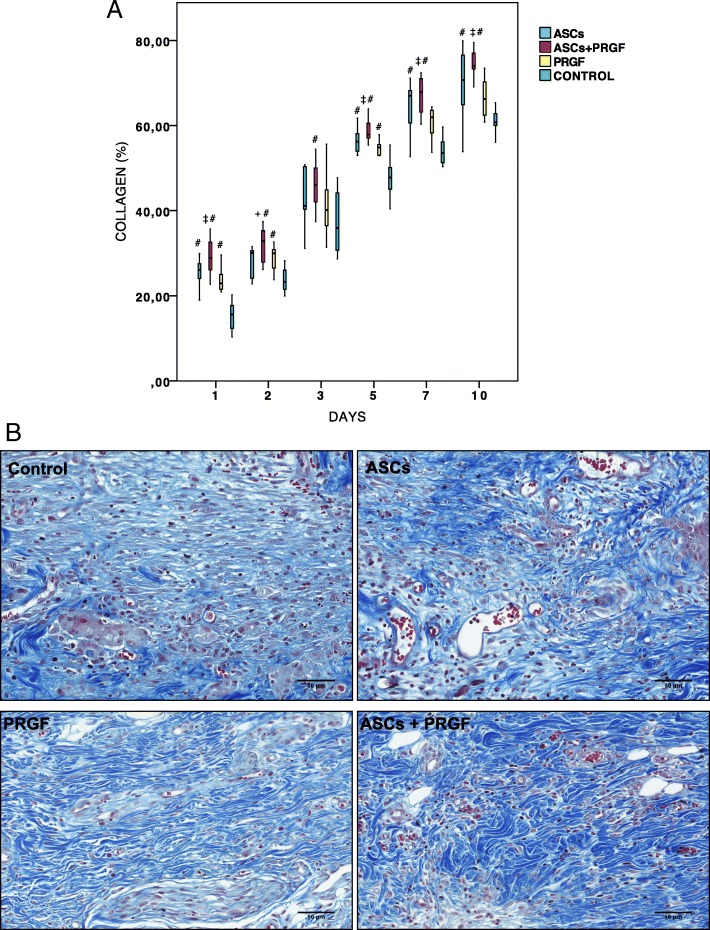
Microscopic wound healing collagen deposition assessment. **a** Quantification of collagen deposition percentage: Comparison in between treatment groups regarding collagen deposition percentage in each of the studied times. Statistical significant differences between groups are shown with the following: + (ASCs group), † (ASCs+PRGF group), ‡ (PRGF group), # (CONTROL group). **b** Masson trichrome staining. Results from a representative animal within the different treatment groups (Scale bar = 10 mm)

Statistical significant differences were obtained in between treatment groups along five checking days except for day 3, appreciating an increase of collagen deposition in ASCs, PRGF and ASCs+PRGF treated wounds with respect to control wounds (*p* = 0.000). Among the treated groups, the highest significant percentage of collagen fibres was shown again by the ASCs+PRGF group not only compared to control groups, but also regarding ASCs treated wounds at day 2 and with PRGF group on days 1, 5, 7 and 10 (Fig. 6).

In the present study, ASCs treated wounds showed significant increased collagen deposition percentages compared to control groups at days 1, 5, 7 and 10.

PRGF infiltrations were found to enhance collagen deposition in our study at days 1, 2 and 5 compared to control groups.

## Discussion

### Wound closure and epithelialization rate evaluation

It has been previously proven that PRGF stimulates cell proliferation, chemotaxis and matrix production [26], and there are several studies that show faster wound healing rates as shown in our study when treated with PRGF [26–28].

A positive correlation was shown between macroscopical and histological results that are in agreement with other published data [13, 26, 28] where both ASCs and PRGF show to enhance reepithelialization and to accelerate wound closure. Furthermore, our results prove that ASCs+PRGF treated wounds offered the best results regarding wound closure and epithelialization. These results are in good agreement with other authors [29] where bone marrow-derived mesenchymal stem cell (BMSCs) plus Plasma rich in Platelets (PRP) treated wounds showed the highest wound healing rate. In this sense, it is known that PRP preserves the viability of MSCs, stimulates their proliferation and phagocytic activity [9].

### Aesthetic assessment of wounds

Paracrine effects of the ASCs seems to be involved in skin rejuvenation [30] providing antiscarring properties [31]. In agreement with our results, no wound retraction after BMSCs injection to full-thickness wounds was observed in mice [32], while other authors [33] observed a positive color improvement in a canine large skin defect treated with allogenic PRP as shown in our study.

Among the growth factors, it has been reported that TGF-ß3 reduces the deposition of collagen during proliferative and remodelling phases, minimizing scar formation [34]. In contrast to the above findings, other studies did not observe significant differences in coloring when PRP was applied probably due to different PRP preparation protocols [35], while in our study significant differences regarding color where observed when PRGF was applied alone or in combination with ASCs compared to control groups (*p* < 0.05).

### Histological inflammatory cell infiltration and distribution evaluation

In our study, despite the anti-inflammatory properties of PRGF and ASCs when applied alone or combined, a more severe inflammatory cell infiltrate was observed at the beginning of the study compared to the last studied days, this could be due, as suggested by other authors, to an earlier activation of the inflammatory phase, limited during a later phase by PRGF and ASCs [29]. In this sense, MSCs coordinate the effects of inflammatory cells and decrease the secretion of proinflammatory cytokines [2].

Previous studies, in agreement with us, have demonstrated that MSCs exert a suppressive effect on local inflammation. A downregulated expression of pro-inflammatory cytokines was exerted by MSCs when applied topically on canine wounds [36]. Interestingly, a reduced inflammatory infiltration in ASCs treated wounds compared to BMSCs and control wounds was observed in a rabbit cutaneous model [37]. Regarding PRP, studies have shown that PRP may inhibit excessive inflammation and interact with macrophages to improve tissue healing [38]. In accordance with our results, some authors [13] observed a complete resolution of the inflammatory process in PRGF treated wounds at day7, while others [39] at day 28.

In line with our results, other authors have proven a synergistic anti-inflammatory effect in BMSCs+PRP group [29].

### Wound healing angiogenesis assessment

Several studies in agreement with our results have shown that the administration of MSCs to acute and chronic wounds improve wound closure by increasing angiogenesis. This process is attributed to a MSCs paracrine signaling as primary mechanism [2]. In line with our results, in a full thickness excisional injury model in rats, ASCs were shown to enhance neovasculogenesis via secretion of VEGF-A, HGF and FGF-2, and validated the differentiation potential of ASCs into endothelial cells [40]. Moreover, a significant increase in angiogenesis was observed in radiation induced ulcers in rats treated with ASCs [41].

Several growth factors are implicated in neovascularisation, including VEGF, bFGF, PDGF and TGF-ß [42]. In concordance with this, enhanced neovascularization was observed, in agreement with our results, by other authors [28] after topical PRP gel application to cutaneous wounds. A significant increased tissue perfusion in PRP-treated flaps and a larger amount of vessels at day 4 was observed in subdermal plexus skin flap in dogs [43]. Moreover, other authors [26, 27] clearly demonstrated that PRGF stimulates angiogenesis.

The combination of ASCs+PRGF showed a synergistic response in terms of angiogenesis in an early phase of the wound healing process, which completely agrees with other author’s findings [44].

### Collagen deposition assessment

The highest percentages of collagen deposition were obtained by ASCs+PRGF treated wounds. These results are in good agreement with other authors [29] which showed that PRP + BMSCs group exhibited a higher density of collagen fibres. Once more in correlation with our results, some authors [45] also obtained higher mean percentage values of collagen in MSCs and MSCs+PRP groups in diabetic mice.

In accordance with our results, MSCs coated sutures showed to enhance collagen deposition in sutured tissues [46]. Suggesting that this could be indirectly mediated by MSCs through the release of soluble factors which stimulate collagen synthesis, or directly through the release of collagen by MSCs. Additionally, higher blue staining density in ASCs treated wounds was observed in rabbit full thickness wounds, revealing new collagen deposition [37].

Regarding the use of PRP in cutaneous wounds and in agreement with our results, an increased mean percentage of collagen fibres in PRP treated wounds was observed in rabbits [28].

## Conclusion

In conclusion, our data suggest that perilesional injection of PRGF, ASCs and PRGF+ASCs have the potential to provide a safe and efficient treatment managing to enhance and shorten wound healing process by accelerating wound closure, epithelialization rate, decreasing inflammatory response, increasing angiogenesis and collagen deposition in acute full-thickness cutaneous wounds mainly during the early wound healing period. In addition, better aesthetic results have also been obtained.

However, among the three treatment choices, the most effective was the ASCs+PRGF group, proving a synergistic effect between PRGF and ASCs.

## Abbreviations

ASCs: Adipose-Derived Mesenchymal Stem Cells
BMSCs: Bone Marrow Mesenchymal Stem Cells
EGF: Epidermal Growth Factor
FGF: Fibroblast Growth Factor
IGF: Insulin-like Growth Factor
KGF: Keratinocytes Growth Factor
MMP-2: Matrix Metalloproteinase
MSCs: Mesenchymal Stem Cells
PDGF: Platelet-Derived Growth Factor
PRGF: Plasma Rich in Growth Factors
PRP: Plasma Rich in Platelets
TGF: Transforming Growth Factor
VEGF: Vascular Endothelial Growth Factor
